# Varying Impacts of MRI-Detected Lesions on Cognitive Function in CADASIL Patients

**DOI:** 10.1192/j.eurpsy.2025.527

**Published:** 2025-08-26

**Authors:** J. H. Park, H. J. Yang, S. H. Lee

**Affiliations:** 1 Psychiatry, Jeju National University Hospital; 2 Psychiatry, Jeju National University, Jeju, Korea, Republic Of

## Abstract

**Introduction:**

Cerebral Autosomal Dominant Arteriopathy with Subcortical Infarcts and Leukoencephalopathy (CADASIL) is a hereditary cerebrovascular disorder caused by point mutations in the NOTCH3 gene located on chromosome 19. The condition is primarily characterized by three core MRI lesions: white matter hyperintensities (WMH), lacunar infarcts, and cerebral microbleeds. Unlike age-related MRI lesions, which can be attributed to multiple factors such as aging, hypertension, and diabetes, the lesions in CADASIL are linked to a single genetic cause, offering a more uniform model for studying the impact of lesion location.

**Objectives:**

This study focuses on CADASIL patients and aims to identify factors associated with dementia while examining the relationships between total WMH volume, WMH location, lacunar infarct count, cerebral microbleeds, and cognitive performance.

**Methods:**

A total of 81 participants were included in the study. Each participant underwent both brain MRI and the CERAD neuropsychological battery. White matter hyperintensity (WMH) volume was assessed via MRI, and the WMHs were categorized by their proximity to the ventricular surface into three types: juxtaventricular (JVWMH), periventricular (PVWMH), and deep white matter hyperintensities (DWMH). DWMH included lesions located beyond 13 mm from the ventricular surface. Additionally, MRI was used to evaluate the number of lacunar infarcts and cerebral microbleeds (CMB) present.

**Results:**

The prevalence of dementia among CADASIL patients was 18.5%. Logistic regression analysis revealed an odds ratio of 1.078 (95% CI = 1.011–1.150) for WMH volume. Similarly, However, the number of lacunar infarcts and CMB did not demonstrate a significant relationship with dementia risk. Linear regression analysis indicated that total WMH volume was significantly linked to performance on several cognitive tests. When WMH volume was categorized by distance from the ventricular surface, JVWMH volume and PVWMH volume werr significantly associated with performance on various cognitve tests. Conversely, DWMH volume did not demonstrate a significant association with cognitive performance in CADASIL patients. The number of lacunar infarcts was correlated with performance on the trail-making test A, the Stroop word test, and the Stroop color test, while cerebral microbleeds did not show significant associations with cognitive performance.

**Conclusions:**

Core MRI-detected lesions exhibit diverse impacts on cognitive function in CADASIL patients. Total WMH volume and JVWMH volume showed a significant correlation with dementia diagnosis. However, DWMH volume did not demonstrate a significant relationship with either dementia diagnosis or specific cognitive functions. The number of lacunar infarcts was notably associated with visual search speed, while CMB were not significantly connected to dementia diagnosis or any particular cognitive functions

**Disclosure of Interest:**

None Declared

